# A qualitative study to assess school nurses' views on vaccinating 12–13 year old school girls against human papillomavirus without parental consent

**DOI:** 10.1186/1471-2458-9-254

**Published:** 2009-07-21

**Authors:** Rebecca Stretch, Rosemary McCann, Stephen A Roberts, Peter Elton, David Baxter, Loretta Brabin

**Affiliations:** 1 Academic Unit of Obstetrics and Gynaecology, University of Manchester, St. Mary's Hospital Research Floor (5th), Oxford Road, Manchester M13 9WL, UK; 2 Greater Manchester Health Protection Unit, Eccles, M30 0NJ, UK; 3 Health Methodology Research Group, University of Manchester, Manchester M13 9PT, UK; 4 Public Health Department, Stockport Primary Care Trust, Stockport SK4 1BS, UK; 5 Public Health Department, Bury Primary Care Trust, Bury M45 7TA, UK

## Abstract

**Background:**

In the UK, parental consent for the routine vaccination of 12–13 year olds schoolgirls against human papillomavirus (HPV) is recommended, although legally girls may be able to consent themselves. As part of a vaccine study conducted ahead of the National HPV Vaccine Programme we sought the views of school nurses on vaccinating girls who did not have parental consent.

**Methods:**

HPV vaccination was offered to all 12 year old girls attending schools in two Primary Care Trusts in Greater Manchester. At the end of the study semi-structured, tape-recorded interviews were conducted with school nurses who had delivered the vaccine (Cervarix™). The interview template was based on concepts derived from the Theory of Planned Behaviour. Transcripts were analysed thematically in order to understand school nurses' intentions to implement vaccination based on an assessment of Gillick competency.

**Results:**

School nurses knew how to assess the competency of under-16s but were still unwilling to vaccinate if parents had refused permission. If parents had not returned the consent form, school nurses were willing to contact parents, and also to negotiate with parents who had refused consent. They seemed unaware that parental involvement required the child's consent to avoid breaking confidentiality. Nurses' attitudes were influenced by the young appearance and age of the school year group rather than an individual's level of maturity. They were also confused about the legal guidelines governing consent. School nurses acknowledged the child's right to vaccination and strongly supported prevention of HPV infection but ultimately believed that it was the parents' right to give consent. Most were themselves parents and shared other parents' concerns about the vaccine's novelty and unknown long-term side effects. Rather than vaccinate without parental consent, school nurses would defer vaccination.

**Conclusion:**

Health providers have a duty of care to girls for whom no parental consent for HPV vaccination has been given, and in the UK, this includes conducting, and acting upon, an assessment of the maturity and competence of an adolescent minor. To facilitate this, policies, training and support structures for health providers should be implemented.

## Background

In September 2008 the Department of Health (DH), the governmental body in the United Kingdom (UK) which provides policy and guidance to the National Health Service (NHS), introduced a primary prevention programme to prevent cervical cancer. This programme offers routine human papillomavirus (HPV) vaccination (Cervarix™, manufactured by GlaxoSmithKline, Rixensart, Belgium) to all 12–13 years old schoolgirls and a catch-up programme for 14 to 18 year olds. HPV is a sexually transmitted infection, but if the vaccine is given prior to the onset of sexual activity, it prevents infection with types 16 and 18 which cause 70% of cervical cancers [1]. The DH recommended that Primary Care Trusts (PCTs), which are the organisations responsible for improving the health of the local population, should deliver the vaccine through schools and all but four of the 152 PCTs have done so. The vaccine is free for the recommended cohorts and PCTs are reimbursed the vaccine and implementation costs.

The age of consent to medical procedures in many countries, including the UK, is 18 years [2,3]. Some countries have enacted laws that permit minors below this age to self-consent although the process is still mediated by the health care provider responsible for deciding whether self-consent is applicable [4]. In the United States the Society for Adolescent Medicine has stated that health care professionals should know and understand the state and federal laws relevant to the delivery of health services to adolescents, and have the skills to apply these legal requirements [5]. In England and Wales, under the 1969 Family Law Reform Act, 16 – 17 years olds can, with some exceptions, consent on their own behalf to treatment. Children below 16 years have a legal right to consent if they have sufficient capacity – generally referred to as 'Gillick competence', following a well-known court case in 1985 [6]. Mrs Gillick challenged her local health authority in order to prevent confidential contraceptive advice being given to under-16 year olds without parental consent. She argued that under-16s did not have legal capacity to give valid consent to contraceptive advice and treatment, and, without parental consent, this was an infringement of parental rights. The Law Lords ruled against Mrs Gillick in 1985. Mr Justice Woolf stated;

"...*whether or not a child is capable of giving the necessary consent will depend on the child's maturity and understanding and the nature of consent required. The child must be capable of making a reasonable assessment of the advantages and disadvantages of the treatment proposed."*

In Scotland the "*Age of Legal Capacity (Scotland) Act 1991" *provides similar legislation, and in Northern Ireland, the House of Lords ruling is assumed to apply. Notably the conditions for self consent do not depend on the chronological age of the child, but the ability to understand the consequences of the intervention. While there is no stated lower limit for assessing a minor's competence to consent to treatment, *The Sexual Offences Act 2003 *states that a child under the age of 13 years is not legally capable of consenting to sexual activity. This does not prevent the provision of confidential advice or treatment for children, but would alert the health practitioner to potential sexual abuse and child protection issues [7]. The "Fraser Guidelines" are a set of specific criteria to be met by medical practitioners when providing contraceptive advice to under 16's without parental knowledge [8]. In addition to assessing competency, these require the health professional to assess the risk to the child of not receiving contraceptive advice, and whenever possible, to persuade the child to involve the parents [9]. Although originally related to contraceptive advice, Fraser guidelines have been widely adopted and applied to the consent process for other sexual health interventions such as treatment of sexually transmitted infections.

In the UK parental consent for HPV vaccination of 12 year olds is sought, although as described above, girls could self-consent. In the information provided to parents the DH advises that the decision is legally the child's but that it is unlikely the injection will be given without parental consent [10]. A previous study of parental acceptability of HPV vaccination reported that, while 80% of parents broadly agreed with HPV vaccination, 44% of vaccine-acceptors would be opposed to providing it without their knowledge [11]. They viewed consent as a parental right and responsibility. Many parents also believe that their child is not, and will not become, sexually active for some years and would prefer a later age of vaccination [12]. If given without their consent, this would involve discussion of sexual issues that parents would prefer to defer till the child is older. Hence applying Gillick competence in a school-based HPV vaccine programme might be both controversial and difficult for school nurses, who administer the vaccine, to implement.

In 2007–08, we undertook a study to assess the feasibility and acceptability of providing HPV vaccination to 12–13 year olds in 36 schools in Greater Manchester, in the northwest of England. As this was a research study parental consent for vaccination was obligatory, but the study provided an opportunity to interview school nurses to ascertain their views on assessing Gillick competence and vaccination of girls whose parents had not given consent.

## Methods

North Manchester NHS Research Ethics Committee approved the study. Cervarix™ was offered at 0, 1, and 6 months to 2817 12–13 year olds between October 2007 and September 2008 in two PCTs in Greater Manchester, the large urban conurbation surrounding the city of Manchester [13]. In July, at the end of the main study, registered nurses working within the NHS school nursing services who had taken part in the study, were invited by the research nurse (RS) for a semi-structured interview. In England, each PCT configures its own delivery plan [14]. In PCT1 four teams of school nurses vaccinated in all secondary schools in their allocated areas. In PCT2, children in all schools were vaccinated by a vaccine team, comprising three school nurses who were helped on the day by the school nurse attached to that particular school. We aimed to interview at least one school nurse from each area team in PCT1 and all nurses on the vaccine team in PCT2. School nurses who assisted the vaccine team in PCT2 were also invited. Recruitment was discontinued when RS considered that little new information was being generated.

Interviews were arranged at a time and place convenient for the school nurse and were recorded using a digital Dictaphone after obtaining written consent. The interview schedule was designed to include concepts referred to in Ajzen's Theory of Planned Behaviour (TPB) [15] and related to the discussion of consent. This framework postulates that actions are motivated by a) attitudes and beliefs – in this case the school nurses' beliefs about the benefits of HPV vaccination; b) subjective norms – perceptions that colleagues and parents would approve of them vaccinating without parental consent; c) perceived control of the action – time constraints and the impact of HPV vaccination policies on their work in schools. This provided a structured set of factors that might explain their intentions to implement vaccination based on assessment of Gillick competency. Interviews were transcribed and analysed using a thematic approach. The interviewer (RS) functioned as the transcriber, verifier and analyst which allowed for the inclusion of notes on relevant non-verbal actions and clarification of mispronunciation or confusing verbal responses. Minor grammatical errors have been corrected when reporting direct quotations. The data were coded to identify distinctive and repetitive themes that were highlighted and categorised according to key concepts loosely corresponding to the TPB. RS and LB both reviewed the data and its analysis to arrive at a consensus on interpretation. No software analysis package was used, nor was member checking employed.

## Results

In total 15 (47%) of the eligible staff took part in the semi-structured interviews, 8 in PCT1 and 7 in PCT2. With the exception of one PCT1 team, at least one nurse from each nursing team was interviewed.

### School nurses' willingness to assess Gillick Competence

All school nurses confidently described procedures for assessing a child's capacity to consent to vaccination. One summary was to:

"...*weigh up in the balance, does this girl understand a) what she is having b) the implication of having it and c) the implication of not having it." *(SN2)

Although they knew what to do, nurses were hesitant to implement vaccination based on this assessment. This applied not only to HPV but also, according to one nurse, to the booster vaccination for tetanus, diphtheria, and oral polio (TdIPV) offered to older children at about age 14 (Year 10) and before age 16. She said:

*"We've introduced Gillick competence for year 10s [TdIPV] for the first time ever and school nurses are really uncomfortable about it." *(SN15)

One school nurse who had worked in youth counselling services, and was more experienced in assessing Gillick competency, thought her colleagues *"...could probably assess but not with a view to give *(doing)*something" *(SN8). Irrespective of their previous experience, every school nurse interviewed said that she would not give HPV vaccination if the parents had returned a refusal form, even if the child was considered to be Gillick competent. If the consent form had not been returned, nurses were generally willing to try and contact the parent for permission, but would still not vaccinate if permission was not forthcoming. Exceptionally, one school nurse stated that if the parent still failed to respond she would vaccinate as *"to me, non-engagement would give me the green light to go ahead and get consent from the child." *(SN5)

### Factors affecting willingness to assess Gillick competency

#### Age

Ignoring the characteristics of individual children, 12–13 year olds were described as very young, immature and easily influenced. They were considered to be *"...still quite vulnerable ... and impressionable" *(SN13), incapable of making a clear and independent judgement about HPV vaccination because they were "*...trusting and easily swayed." *(SN4) In contrast with the view expressed above that school nurses were uncomfortable with giving the TdIPV on the basis of self consent, five nurses would have been "...*happier if they were year 10s *[age 14–15] *rather than Year 8s *[age 12–13] " as they were older, more mature, and therefore considered more competent.

The physical appearance of 12–13 year olds also influenced school nurses' views. One stated:

*"Some of them are still little children and haven't started to develop and yet some of them have gone through puberty and look like women." *(SN6)

Another said,

*"You get some very mature but some look very tiny." *(SN12)

As well as general behaviour and appearance, this was thought to be *"a very borderline age" *for assessing competency. (SN8) This apprehension pertained to the '*legalities' *of treating under-13s. One noted,

*"There's the under-13's Special Act about consent... The Law says that under 12 or 13 they have not got the ability to understand and cannot consent." *(SN14)

This statement was misquoted as the Act refers to the fact that children under 13 cannot consent to sexual activity. Similarly the context for applying Fraser guidelines, which are specific to sexual health treatment, was often misunderstood. This was illustrated in the following statement:

"*We are using Fraser guidelines, which started off being on contraception and I know we can use it in all realms." *(SN13)

#### Belief in vaccination programmes

Childhood vaccination was deemed an important, effective, and worthwhile intervention, and described as *"wonderful," *(SN2) "*brilliant," *(SN8) *"fantastic" *(SN9) and "*vital to the health of young people." *(SN4) Nurses deplored the *"furore" *(SN11) surrounding the measles, mumps and rubella vaccination which had reduced uptake rates and had led to measles outbreaks. Despite believing that parents had a responsibility to have their child vaccinated, both to protect the child and the wider society, four nurses commented that the views of people who did not agree with vaccination should be respected.

#### Attitudes to HPV vaccination

Nurses expressed positive attitudes to the HPV vaccine. Many made comments such as,

"*I thought in principle it was great" *(SN 2) and, "*I think it's super – one we should embrace." *(SN5)

In practice there was some conflict between their professional and personal beliefs which were less accepting of HPV vaccination. The majority of school nurses expressed some reservations about vaccinating their own child and just four stated immediately that they would consent. One of them recognised this tension, saying,

"*I do think you wear very different hats when you're a professional and when you're in a personal situation." *(SN16)

Another said,

*"I can't justify vaccinating other people's children if I didn't think it was suitable for my own child." *(SN8)

Their main concerns were unknown "*long-term complications" *(SN13) and the novelty of the vaccine which made it *"controversial." *(SN12) Their comments suggested that most would have consented eventually to their own children being vaccinated. One noted,

*"I think I might well have said 'yes' in the end but I would have done a heck of a lot of research beforehand"*. (SN2)

Two nurses had cared for patients with severe cervical dysplasia and having seen *"the trauma they have been through" *(SN12) had decided that the benefits of preventing HPV infection outweighed the risk of possible long-term side effects.

#### Views on the rights of parents

Although school nurses acknowledged a child's right to be vaccinated and their own duty to protect children from infectious diseases, they still concurred with the view that parents had a right to refuse the vaccination. This was based on the parent's overarching responsibility for a child "*under the legal age." *(SN 6) They also empathised with parents, acknowledging how they would feel if their child were vaccinated against their wishes. They made comments such as,

"*My daughter is a very young 12 year-old and as a parent I would not be happy" *(SN16) or,

*"I would be absolutely devastated and angry if someone went against my wish." *(SN17)

By anticipating their own reactions, they became conscious of the possible repercussions from other parents if they vaccinated without consent. Nor did they wish to break the *"trust of the parents" *(SN17) as they might need to deal with the same parents in the future (especially in the context of child protection). Going against parental wishes could jeopardise co-operation. One concluded that *"we have to abide by the parents wishes" *(SN6) and another summarised a common view as follows:

"*I value the parent's opinion first." *(SN12)

It was thought unlikely that girls of this age would challenge parental wishes. School nurses did not indicate any intention to routinely assess Gillick competence for non-consented girls. If such a child expressed an interest in vaccination, nurses said that their first response would be to contact the parents and try to reach a consensus. The confidentiality of the child in this matter was not considered although one school nurse said she would obtain permission from the child before contacting parents. Another acknowledged the dilemma between contacting the parents to discuss the child's request and breaching a child's right to confidentiality. If agreement with parents could not be reached, since HPV vaccination was not seen as an urgent intervention, their proposed solution was to delay vaccination until the girl was older. One said,

*"I know the younger they have it the better, but there isn't the same degree of urgency *(for HPV)* and I would far rather sort it out and have everybody happy." *(SN2)

### Practical barriers to assessing Gillick Competency

Barriers such as the difficulty of assessing competency due to time constraints and limited space for private discussion at a vaccination session were mentioned. School nurses were also concerned that the girls would not provide a reliable medical history, particularly adverse events associated with previous vaccinations, which presented a clinical risk.

## Discussion

Despite strong beliefs in childhood vaccination and the benefits of preventing HPV infection, these school nurses would not vaccinate 12 year old girls without parental consent. They were hesitant to assess Gillick competence due to confusion about laws and medical guidelines, the young age of girls and to some extent, their personal reservations as parents which made them sympathetic to parental concerns about vaccine safety and efficacy. School nurses can acknowledge and try to address parental concerns but ultimately have a duty of care and a legal obligation to determine whether girls whose parents have refused, or not responded to, a vaccine invitation are competent to consent in their own right.

This paper provides an example of the current confusion surrounding the legal framework for adolescent consent which applies not just in the UK, but more broadly [16-18]. A case study in Australia, involving under 18's with anorexia nervosa, revealed a wide discrepancy in general practitioners' understanding and implementation of current legal principles [16]. Their interpretation of the same medical, legal and patient-related factors was diverse and threatened to impinge on young people's rights to health care [18]. In our study, nurses were unclear which guidelines to apply. For example they perceived that vaccination against a sexually transmitted infection could be construed as a sexual health intervention, in which case Fraser guidelines could apply and girls would be encouraged to talk to their parents. The national vaccine programme has, however, presented HPV vaccination as a cervical cancer prevention strategy (applying Gillick competence without requiring parental consultation) as most girls will not have been sexually exposed to HPV infection at this age. This young age suggested to some nurses that girls would not be competent to self-consent, reflecting that chronological age is a disputed predicator of an individual's maturity [19] Brazier [20] claimed that children below 12 years never, and 12–14 year olds only rarely, attained sufficient maturity to be able to consent whereas other researchers [21] regarded 12-year olds as competent. Children believed themselves to have sufficient maturity and understanding to consent to immunisation at a mean of 12.3 years [22]. A study among young people considering elective surgery reported that girls could make a reasoned decision at 13.1 years (mean) and that even children below ten years could comprehend the treatment proposed and the consequences of surgery [23]. Children usually receive more information the older they are [24], resulting automatically in them being assessed as competent according to age. Studies to date indicate that assumptions about age and immaturity are ubiquitous and not sufficiently addressed in health worker training.

To our knowledge, this paper is the first in the UK to report on the views of school nurses involved in the implementation of a school-based HPV vaccine programme but this research has a number of limitations. Firstly, school nurses were involved in a study in which ethics approval required written parental consent to HPV vaccination whereas under the national programme, parental consent is not mandated. Secondly, qualitative research cannot be generalised. Although these nurses were broadly representative of the two PCT school nursing teams, their views may not represent those of all school nurses. Thirdly, as experience in delivering HPV vaccination increases, school nurses may feel more confident to assess individual girls. Despite these limitations, this research highlights some of the challenges facing school nurses. A probable prerequisite for influencing parents who have vaccine safety concerns is a trusting and respectful relationship between the health care provider and parent [25] and complying with the parents' wishes may increase the possibility of future consent. In doing so, however, the duty of confidentiality owed to a child who does not wish her parents to know she wishes to be vaccinated, as well as the duty to pro-actively establish the wishes of non-consented girls, may be overridden. This could damage trust between the child and nurse and prevent the child requesting help in the future [26].

## Conclusion

An important role for health professionals working with children is to develop the child's decision-making competence [16]. In the UK, HPV vaccination is just one of the roles of the school nurse who relies on maintaining good relations with schools and parents. In practice it may be very difficult to provide confidential HPV vaccination to a Gillick competent child in the school setting. Girls who request vaccination following parental refusal may have to be offered vaccination at a different venue where confidentiality and medical follow-up can be assured. In all health settings where HPV vaccination is provided, health workers must have a clearer understanding of the relevant legal and medical guidelines, and follow a structured approach to assessing competency. To encourage staff to undertake assessment more readily also requires clear policy, managerial and legal support structures to be in place in the event of parental complaint.

## Competing interests

RS has received honoraria for meetings organised by GlaxoSmithKline (GSK). LB has received similar fees as well as travel and conference costs from both GSK and Merck. The study was funded by GSK.

## Authors' contributions

RS managed the study, collected the data and drafted the paper; LB conceived the study and finalised the paper; SR was responsible for the design of the main study of which this formed a part and he reviewed the final draft; PE and DB helped to co-ordinate the study within their Primary Care Trusts. All authors read and approved the manuscript.

## Pre-publication history

The pre-publication history for this paper can be accessed here:


